# PPAR‐γ‐induced changes in visceral fat and adiponectin levels are associated with improvement of steatohepatitis in patients with NASH

**DOI:** 10.1111/liv.15005

**Published:** 2021-07-21

**Authors:** Amalia Gastaldelli, Silvia Sabatini, Fabrizia Carli, Melania Gaggini, Fernando Bril, Renata Belfort‐DeAguiar, Vincenzo Positano, Diana Barb, Sushma Kadiyala, Stephen Harrison, Kenneth Cusi

**Affiliations:** ^1^ Diabetes Division The University of Texas Health Science Center at San Antonio San Antonio TX USA; ^2^ Institute of Clinical Physiology National Research Council CNR Pisa Italy; ^3^ Università degli Studi di Siena Siena Italy; ^4^ Division of Endocrinology, Diabetes and Metabolism University of Florida Gainesville FL USA; ^5^ Department of Internal Medicine and Endocrinology Yale University School of Medicine New Haven CT USA; ^6^ Fondazione Toscana Gabriele Monasterio Pisa Italy; ^7^ Division of Endocrinology, Diabetes and Metabolism Malcom Randall Veteran Administration Medical Center at Gainesville Gainesville FL USA; ^8^ Pinnacle Clinical Research Group San Antonio TX USA

**Keywords:** adiponectin, fatty liver, insulin resistance, NASH, pioglitazone, PPAR‐y, type 2 diabetes mellitus, visceral fat

## Abstract

**Background and Aims:**

Peroxisome proliferator‐activated receptor (PPAR)‐γ agonists decrease hepatic/visceral fat (VF) and improve necroinflammation despite subcutaneous (SC) fat weight‐gain. Understanding the impact of changes in VF, VF‐to‐SC fat distribution (VF/SC) and adiponectin (ADPN) levels in relation to histological improvement after weight‐loss or pioglitazone is relevant as novel PPAR‐γ agonists are being developed for treating non‐alcoholic steatohepatitis (NASH).

**Methods:**

Fifty‐five patients with NASH received a −500 kcal/d hypocaloric diet and were randomized (double‐blind) to pioglitazone (45 mg/d) or placebo for 6‐months. Before and after treatment patients underwent a liver biopsy and measurement of hepatic/peripheral glucose fluxes, hepatic/adipose tissue‐IR and, in 35 patients, hepatic and VF/SC‐fat was measured by magnetic resonance spectroscopy/imaging. Data were examined by multivariable statistical analyses combined with machine‐learning techniques (partial least square discriminant analysis [PLS‐DA]).

**Results:**

Both pioglitazone (despite weight‐gain) and placebo (if weight‐loss) reduced steatosis but only pioglitazone ameliorated necroinflammation. Using machine‐learning PLS‐DA showed that the treatment differences induced by a PPAR‐γ agonist vs placebo on metabolic variables and liver histology could be best explained by the increase in ADPN and a decrease in VF/SC, and to a lesser degree, improvement in oral glucose tolerance test‐glucose concentrations and ALT. Decrease in steatosis and disease activity score (ballooning plus lobular inflammation) kept a close relationship with an increase in ADPN (*r* = −.71 and *r* = −.44, *P* < .007, respectively) and reduction in VF/SC fat (*r* = .41 and *r* = .37, *P* < .03 respectively).

**Conclusions:**

Reduction in VF and improved VF/SC‐distribution, combined with an increase in ADPN, mediate the histological benefits of PPAR‐γ action, highlighting the central role of fat metabolism and its distribution on steatohepatitis disease activity in patients with NASH.

**Abbreviations**
Adipo‐IR: adipose tissue insulin resistance index
AS: Activity Score
Hep‐ISI: hepatic insulin sensitivity index
IL‐6: interleukin 6 concentration
IR: insulin resistance
NAFLD: non‐alcoholic fatty liver disease
NAS: NAFLD Activity Score
NASH: non‐alcoholic steatohepatitis
OGIS: oral glucose insulin sensitivity index
Periph‐ISI: peripheral insulin sensitivity index
PLS‐DA: partial least square discriminant analysis
PPAR: peroxisome proliferator‐activated receptor
SC: subcutaneous fat
T2D: type 2 diabetes
TGF‐β: transforming growth factor‐β concentration
TNF‐α: tumour necrosis factor‐α concentration
VF: visceral fat
VF/SC: visceral‐to‐subcutaneous fat ratio

**Key points**

- The mechanisms leading to a reduction of liver fat accumulation and amelioration of steatohepatitis disease activity (ballooning and inflammation) with the peroxisome proliferator‐activated receptor (PPAR)‐γ agonist pioglitazone remain unclear.
- By using a machine learning approach and partial least square discriminant analysis (PLS‐DA) to discriminate the effect of pioglitazone treatment, we were able to link improvement in hepatic metabolism and in steatohepatitis disease activity to the increase in plasma adiponectin (ADPN) levels and the decrease in the visceral‐to‐subcutaneous fat ratio (VF/SC), and to a lesser degree, improvement in plasma glucose during an oral glucose tolerance test and ALT levels.
- These findings provide provocative new knowledge that the significant reversal of “lipotoxicity” by PPAR‐y agonist treatment in patients with non‐alcoholic steatohepatitis is strongly linked to adipose tissue re‐distribution (VF/SC) and improvement in adipose tissue function (ie increase in plasma ADPN).

## 1 INTRODUCTION

The incidence of obesity and non‐alcoholic fatty liver disease (NAFLD), which includes both hepatic steatosis and non‐alcoholic steatohepatitis (NASH), is rapidly increasing.^1^ Excessive intrahepatic triglyceride content (“liver fat”) and visceral fat (VF), more than total amount of fat, have been suggested as the major determinants of metabolic dysregulation,^2^, ^3^, ^4^, ^5^, ^6^ but clinical longitudinal data are lacking.

It is well‐established that increased waist circumference is a major risk factor for NASH, especially in lean NAFLD,^7^ that have been reported to have also increased VF compared to subjects without NAFLD.^8^ Several groups have shown that VF is strongly correlated with liver fat^9^, ^10^, ^11^ and with increased hepatic gluconeogenesis and hepatic insulin resistance (IR).^9^, ^12^, ^13^ Moreover, both obesity and diabetes are conditions associated to increased VF, hepatic fat, IR and steatohepatitis.^4^, ^5^, ^6^, ^8^, ^9^, ^11^, ^14^, ^15^

NAFLD is also associated with IR in tissues other than liver, like muscle and adipose tissue.^9^, ^15^, ^16^ Bril et al^16^ have shown that even low accumulation of hepatic TG (between 2% and 5%) is associated with reduced insulin sensitivity in liver, muscle and adipose tissue. On the other side, there is no linear increase in inflammation, ballooning or fibrosis with intrahepatic triglyceride content.^16^ Adipose tissue IR is a major risk factor for both severity of liver disease,^17^, ^18^ as well as for type 2 diabetes (T2D),^19^ at least in part as excess lipolysis associated with adipose tissue IR cause an overflow of fatty acids from the periphery to the liver that can worsen disease activity (ballooning and lobular inflammation). We have shown that the accumulation of intrahepatic triglycerides and of VF increase the risk of metabolic derangements in patients with or without NAFLD.^15^ This occurs in a step‐wise manner and is associated with an increase in hepatic, muscle and adipose tissue IR starting with the least metabolic harm in the group with “low Liver Fat/low VF” followed by the group with “low Liver Fat/high VF”, “high Liver Fat/low VF” and with most severe IR in the “high Liver Fat/high VF” group, suggesting a potential greater role for VF than previously appreciated.^10^ A recent study by White et al^20^ has renewed the interest on the role of VF during pioglitazone, as they reported that improved insulin sensitivity in non‐diabetic healthy women was associated with a significant decrease in VF and a predominant increase in the formation of new adipocytes from metabolically protective lower‐body fat depots (gluteal and femoral), rather than from metabolically harmful abdominal fat depots.

While peroxisome proliferator‐activated receptor‐gamma (PPAR‐γ) agonists promote weight gain (mainly as subcutaneous [SC] fat tissue), they also decrease both hepatic steatosis and VF^13^, ^21^, ^22^, ^23^ and improve peripheral,^13^, ^24^ adipose tissue^25^, ^26^, ^27^ and liver^22^, ^28^ insulin sensitivity. A decrease in liver IR with pioglitazone is associated with lower rates of hepatic gluconeogenesis driven, at least in part, by a reduction in the VF depot.^28^, ^29^ Although no drug has been approved yet for the treatment of NASH, there is clearly a role for PPAR‐γ agonists given the positive effect of pioglitazone in patients with biopsy‐proven NASH^21^, ^22^, ^23^, ^30^, ^31^, ^32^ and its incorporation into several guidelines,^33^, ^34^, ^35^ as well as novel PPAR‐γ agonists being tested in patients with NASH.^36^ Therefore, it is of critical clinical relevance to understand the precise mechanism of action of PPAR‐γ agonists in NASH.

We hypothesized that a reduction of VF (or the VF/SC distribution ratio) together with the improvement in adipose tissue metabolism, play a significant role in the reduction in liver fat and amelioration of ballooning and lobular inflammation during either weight loss or PPAR‐γ agonist treatment. However, it has been difficult to separate the tangled web of metabolic factors at play associated with treatment. To this end, we used a novel machine learning approach with partial least square discriminant analysis (PLS‐DA),^37^ a methodology that can manage simultaneously a large number of highly intercorrelated predictors. With this more in‐depth novel approach we conducted a post‐hoc analysis of subjects with biopsy‐proven NASH randomized to either pioglitazone or placebo treatment for 6 months to study the individual and combined impact of changes in fat distribution (SC vs VF), adipose tissue IR, and plasma adiponectin (ADPN) concentration, relative to the improvement in disease activity on histology in patients with NASH.

## 2 METHODS

This is a post‐hoc analysis of a previously published study^22^ (clinical trial registration number NCT00227110) that examined the role of pioglitazone (45 mg/d for 6 months) vs placebo with dietary intervention in the treatment of NASH in patients with glucose intolerance (IGT) or T2D. The CONSORT 2010 Flow Diagram is included under supplemental data. The data on body fat distribution are new and never published. All subjects gave written informed consent prior to participation. All authors had full access to all of the data in this study and I take responsibility for the integrity of the data and the accuracy of the data analysis.

### 2.1 Study subjects

The analyses were performed on patients with biopsy‐proven NASH that completed the trial with a biopsy and with complete data on liver fat and VF measured by MRI/MRS before and after treatment, that is, 35 of the 47 subjects (74%) from the initial cohort,^22^ see Consort‐flow diagram in Supplementary Material. After baseline metabolic measurements, patients were randomized to either oral placebo or pioglitazone (ACTOS^®^; Takeda Pharmaceuticals) 30 mg/d, titrated after 2 months to 45 mg/d until the end of the 6‐month study. The cohort included both patients with T2D or IGT diagnosed by an oral glucose tolerance test (OGTT) performed prior to enrolment and end of study. Liver biopsies were scored according to Kleiner et al.^38^

Patients on placebo were divided into two groups: the first group included those that after six months showed a reduction in body weight (BW) of at least 3% of the initial BW (n = 8; “BW‐loss”; Table 1) and those in whom weight did not change (ie, weight loss <3%) or increased their weight (n = 10; “Diet‐Fail”). The 3% cut‐off was decided based on previously published analyses.^39^

**TABLE 1:** Patient characteristics and changes in clinical and laboratory parameters with pioglitazone vs placebo

	Placebo BW‐loss		*P* value	Placebo diet‐fail		*P* value	Pioglitazone	
	Pre N = 8	Post	Change vs PIO	Pre N = 10	Post	Change vs PIO	Pre N = 17	Post
No‐Diab/T2D	7/1	8/0		4/6	3/7		8/9	12/5
Age	52 ± 3		ns	51 ± 4		Ns	51 ± 2	
BMI	32.0 ± 1.5	30.7 ± 1.5*	<.0001	32.6 ± 1.1	33.3 ± 1.2	Ns	32.9 ± 1.0	34.6 ± 1.2*
Body weight (kg)	88.4 ± 5.3	84.8 ± 5.1*	<.0001	88.8 ± 4.2	90.6 ± 4.2	Ns	92.0 ± 3.7	96.8 ± 4.3*
Body fat (kg)	32.1 ± 2.7	30.2 ± 2.5*	<.0005	31.3 ± 2.7	31.9 ± 2.8	Ns	32.7 ± 2.2	34.8 ± 2.3*
Waist (cm)	88.4 ± 15	84.8 ± 14.4	<.0001	88.8 ± 13.4	90.6 ± 13.3	Ns	92.0 ± 15.4	96.8 ± 17.9
A1c (%)	5.6 ± 0.3	5.6 ± 0.2	<.02	6.4 ± 0.4	6.3 ± 0.3	<.007	6.2 ± 0.4	5.6 ± 0.2*
FPG (mmol/L)	5.4 ± 0.1	5.4 ± 0.1	<.01	6.7 ± 0.45	7.0 ± 0.5	<.02	6.5 ± 0.5	5.6 ± 0.2*
FPI (pmol/L)	77 [43]	71 [72]	ns	77 [63]	99 [56]	<.02	89 [60]	42 [45]*
AST (U/L)	43 ± 5	35 ± 7	ns	31 ± 2	31 ± 2	<.006	42 ± 3	27 ± 1*
ALT (U/L)	57 ± 9	44 ± 9	.02	42 ± 7	38 ± 6	<.0007	58 ± 5	28 ± 2*
FFA (µmol/L)	648 ± 54	630 ± 81	.03	621 ± 58	789 ± 56	.01	790 ± 54	606 ± 70*
Adiponectin (µg/mL)	9.6 ± 1.5	10.1 ± 1.7	<.0002	6.3 ± 1.2	6.0 ± 1.2	<.0001	6.5 ± 0.6	15.6 ± 1.4*
TNF‐α (pg/mL)	1.72 ± 0.70	1.91 ± 0.61	.036	1.78 ± 0.78	1.74 ± 0.72	Ns	2.33 ± 0.95	2.15 ± 0.82
IL‐6 (pg/mL)	3.64 ± 2.02	3.43 ± 2.37	ns	3.04 ± 1.91	2.48 ± 1.25	Ns	3.39 ± 1.01	3.17 ± 2.06
TGF‐ß (ng/mL)	17.33 ± 6.59	19.68 ± 6.35	ns	22.89 ± 10.75	23.33 ± 11.45	Ns	31.11 ± 10.17	23.68 ± 8.19*
OGIS (mL/min·kg)	4.0 ± 0.1	3.9 ± 0.2	.02	3.9 ± 0.2	3.8 ± 0.1	<.006	3.9 ± 0.2	4.6 ± 0.2*
Adipo‐IR (mM·pM)	53 [15]	58 [38]	.03	45 [28]	69 [37]	<.008	68 [37]	26 [23]*
HepIS (µmol/min·kg·nM)^−1^	1.25 [0.70]	1.62 [2.26]	ns	1.07 [1.06]	1.14 [0.67]	.02	1.18 [1.15]	2.45 [1.85]*

Note

Average ± SE if normally distributed, or median [interquartile range] if not normally distributed.

Adipo‐IR, adipose tissue insulin resistance index; ALT, alanine transaminase; FFA, free fatty acid; IL‐6, Interleukin‐6; OGIS, oral glucose insulin sensitivity index; TGF‐ß, transforming growth factor beta; TNF‐α, tumour necrosis factor alpha.

* *P* < .05 vs pre.

### 2.2 Study protocol

During the 4‐week run‐in period, subjects were instructed by the research dietician not to change their caloric intake or physical activity and then educated to reduce their intake by −500 kcal/d that was monitored during follow‐up visits.^22^ Baseline and end‐of‐treatment metabolic measurements included: (a) fasting plasma glucose, HbA_1c_, lipid profile, insulin, free fatty acid (FFA), ADPN, tumour necrosis factor alpha (TNF‐α), Interleukin‐6 (IL‐6), Transforming growth factor beta (TGF‐β), C‐reactive protein (CRP, available for n = 7 Pioglitazone and n = 4 placebo treated subjects) concentrations; (b) liver fat content by magnetic resonance spectroscopy (^1^H‐MRS) and visceral and SC fat by MR‐Imaging; (c) Liver biopsy; (d) OGTT (75g+[1‐^14^C]‐glucose) with [3‐^3^H]‐glucose infusion for the determination of glucose fluxes.^22^

### 2.3 Body fat distribution

^1^H‐MR spectra of the liver and visceral and SC fat images were acquired using a 1.9T MR scanner (Elscint Prestige Ltd.). VF and SC were measured as published previously^40^: briefly, 32 transverse, T1‐weighted images centred through the space between L4 and L5 were acquired in breath hold. Visceral and SC fat volumes were measured from MRI images using the Hippo Fat software, and a factor of 0.92 was used to convert adipose tissue volume into adipose tissue mass.^40^

### 2.4 Modelling and statistical analysis

Analysis of metabolic fluxes **(**Fluxomics)^9^, ^41^ included the measurement of endogenous glucose production (EGP, µmol·min^−1^·kg^−1^), peripheral glucose clearance, hepatic insulin sensitivity index (Hep‐ISI), as the inverse of (EGP × Ins), peripheral insulin sensitivity during OGTT oral glucose insulin sensitivity index (OGIS),^40^ adipose tissue IR index (Adipo‐IR) calculated as (FFA × Ins, mM·pM).^9^, ^19^

We used a machine learning approach to discriminate between pioglitazone vs placebo, considering also the effect of weight loss that occurred during placebo intervention (PIO vs “BW‐loss” or “Diet fail”) and to evaluate the predictive power of metabolic changes on the improvements in histology, in particular on steatosis, activity score (AS = ballooning + lobular inflammation,^42^), and NAFLD activity score (NAS), that is a combination of the two. Data were analysed using PLS‐DA^37^ a classification method widely adopted in chemometrics based on linear transformations of features that can manage simultaneously a large number of predictors, even with a small number of subjects. PLS‐DA is also able to determine the discriminative power of each variable; this is particularly useful when predictors are highly intercorrelated and it is hard to identify the features primarily associated to the classification factor. PLS‐DA’s performance and accuracy in prediction can be assessed with statistical measurements, ensuring its goodness with respect to random classifications. We used as predictors metabolic and anthropometric data previously transformed as log_2_(post/pre), indicating fold changes above baseline, and then mean‐centred and scaled to unit variance. Simple differences (post–pre) of the histological parameters were added (and scaled) to the models on treatment, whereas treatment was used as a covariate in models based on histological differences (Tables S3 and S4). PLS‐DA method is also able to rank the resulting statistical models based on accuracy in prediction evaluated through the mean misclassification error rate (obtained by seven‐folds cross‐validation which was repeated fifty times in order to manage random anomalies because of particular splits). Accuracy was also used to tune the best number of components of each model. Accordingly, final PLS‐DA models were built on the entire dataset and the Variable Importance in Projection (VIP) score used as measure to assess the discriminatory power of features with the cut‐off >1. Random permutation tests were finally run with 1000 permutation to assess the statistical significance of our models.

Multivariable linear regression analysis was used to estimate associations among changes in continuous variables first in single groups and then in the whole dataset (Spearman rank test).

Data in tables and figures are given as the mean ± SE if normally distributed, as median and interquartile range (in square brackets) if skewed distribution. Group differences were analysed by Mann‐Whitney test and Kruskal‐Wallis test for binary and multiple comparison respectively. A two‐tailed *P* < .05 was considered statistically significant.

Missing data were imputed using K‐nearest neighbours’ method (KNN).

## 3 RESULTS

**FIGURE 1:**
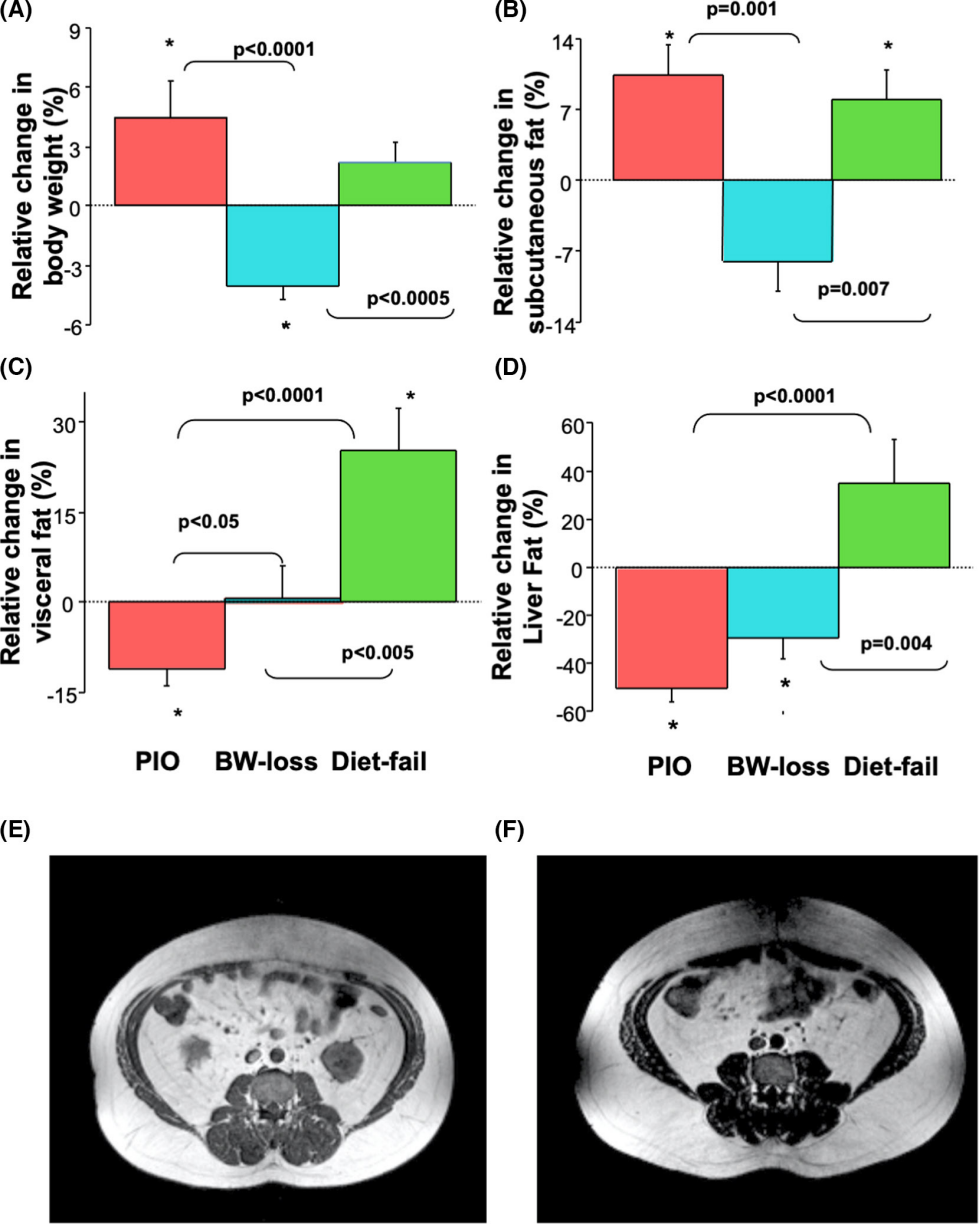
Changes in clinical parameters in patients with NASH after pioglitazone (“PIO”; blue bars), weight loss following dietary counselling (“BW‐loss”; red bars), or dietary failure to nutritional counselling (“Diet‐fail”; green bars). Panel A: body weight (BW); Panel B: Subcutaneous adipose tissue; Panel C: Visceral fat; Panel D: Liver fat *P < .05 vs pretreatment. MRI scan of a study patient before (Panel E) and after (Panel F) treatment with pioglitazone. Clinical characteristics: 39 year old male with type 2 diabetes: Fasting plasma glucose decreased from 8.8 to 7.0 mM and HbA1c from 7.9% to 6.2%; BMI increased from 35.6 to 37.3 kg/m^2^. The ratio of visceral‐to‐subcutaneous fat distribution (VF/SC) decreased from 0.54 to 0.36, while liver fat decreased from 8.4% to 2.5%

Patients were divided into three groups: those randomized to pioglitazone, those on placebo who showed a decrease in BW from baseline (“BW‐loss”), and those that failed to succeed in losing weight or increased their BW (“Diet‐Fail”). Patients had similar clinical and laboratory variables at baseline (Table 1), except for a slightly lower, fasting plasma glucose concentration in the BW‐loss vs Diet‐Fail (*P* = .002) and pioglitazone groups (*P* = ns). Patients under pioglitazone treatment gained on average 4.8 ± 0.7 kg (+5%; *P* = .04 vs baseline; *P* < .0001 vs BW‐loss; ns vs Diet‐Fail). Patients in the placebo in the BW‐loss group lost on average −3.6 ± 0.6 kg (−4%; *P* = .001 vs baseline) compared to dietary intervention failures (Diet‐Fail) that gained 1.9 ± 0.9 kg (+2%; *P* = ns vs baseline) (Figure 1A).

Compared to baseline (week 0), only patients treated with pioglitazone had a reduction in plasma ALT levels, (*P* < .0001) and AST (*P* < .001), with no significant change in placebo groups (Table 1). Also as shown in Table 1, the placebo groups had no significant improvement in metabolic parameters, although the BW‐loss group showed a trend towards improvement. On the other hand, pioglitazone led to an improvement in all indexes of IR, particularly in the adipose tissue showing a reduction in Adipo‐IR and an increase in ADPN concentrations. Hepatic‐ISI and peripheral insulin sensitivity index (Periph‐ISI) significantly increased in the pioglitazone group compared to both BW‐loss and Diet‐Fail. Among inflammation markers, TGF‐β was significantly reduced (*P* = .02) and TFN‐α, IL‐6 showed a trend towards improvement in pioglitazone group, while no significant changes were observed in the placebo group (Table 1). Although CRP was measured only in a small subgroup, we observed a significant decrease in PIO vs placebo at 6 months (post‐pre −3.1 ± 1.6 vs +1.0 ± 0.5 mg/L, *P* = .01).

In agreement with previous studies, weight gain with pioglitazone was largely because of an increase in abdominal SC fat (pioglitazone: +10%, *P* = .004 vs baseline; vs −8% in BW‐loss, *P* = .001) (Figure 1B); despite weight gain, patients receiving pioglitazone had a reduction in VF of 11%, which was greater than in the BW‐loss (0%, Figure 1C). Compared to baseline, Diet‐Fail patients gained liver fat while both pioglitazone (−50% *P* < .0001) and BW‐loss (−29% *P* = .01) had a significant decrease in liver fat (Figure 1D). Figure 1E,F are the MRI abdominal images of one of the subjects treated with PIO where it is evident the increase in SC fat and the decrease in VF. In contrast, Diet‐Fail group gained VF, consistent with the overall weight gain.

### 3.1 Selection of variables that better explained the effect of treatment

As shown in Table 1, treatment with dietary intervention plus placebo vs pioglitazone induced several and different changes on glucose and lipid metabolism and liver histology. These also included the improvements in adipose tissue function and IR as shown by the increase in ADPN and the decrease in TGF‐β and TNF‐α, although IL‐6 did not change. However, most of the changes were highly intercorrelated. Thus, to identify which changes are mainly associated with treatment, we adopted a machine learning approach combined with PLS‐DA analysis. Compared to traditional approaches, this method has the advantages of evaluating the metabolic differences between the overall effects of treatments while selecting the features that better explained those differences.

**FIGURE 2:**
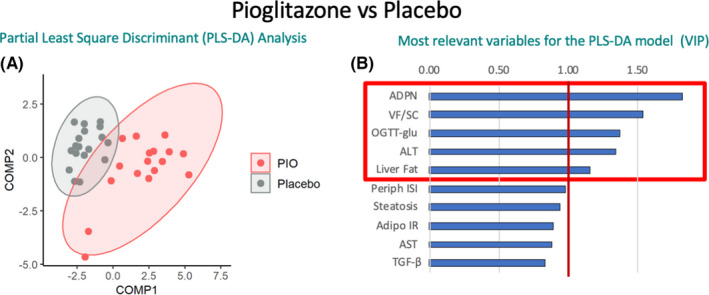
Machine learning approach with partial least square discriminant analysis (PLS‐DA) to discriminate the effect of pioglitazone (PIO) vs placebo (either “BW‐loss” or “Diet fail”), using all metabolic (log2 post/pre) and histological (post‐pre) variables. PLS‐DA is able to determine the discriminative power of each variable, improving model prediction. Panel A: Scores plot of PLS‐DA in PIO (red points) vs placebo (grey points) subjects’ classification. Panel B: Variables contribution to the PLS‐DA model, measured through VIP index. The red box features variable with VIP > 1 that are considered relevant in the discrimination. Adipo‐IR, adipose tissue IR index; ADPN, adiponectin; ALT, alanine transaminase; ballooning, Ballooning score in liver biopsy; OGTT‐glu, mean glucose concentration during OGTT; Periph ISI, peripheral insulin sensitivity index calculated as OGIS

We applied PLS‐DA to discriminate subjects treated with dietary intervention plus placebo vs pioglitazone (Figure 2), and the subgroup that lost weight in the placebo group (“BW‐loss”) vs pioglitazone (Figure S1). The classification models had good performances with significant *P*‐values (*P* < .01 by permutation test) and high accuracy (detailed in Tables S1 and S2).

**FIGURE 3:**
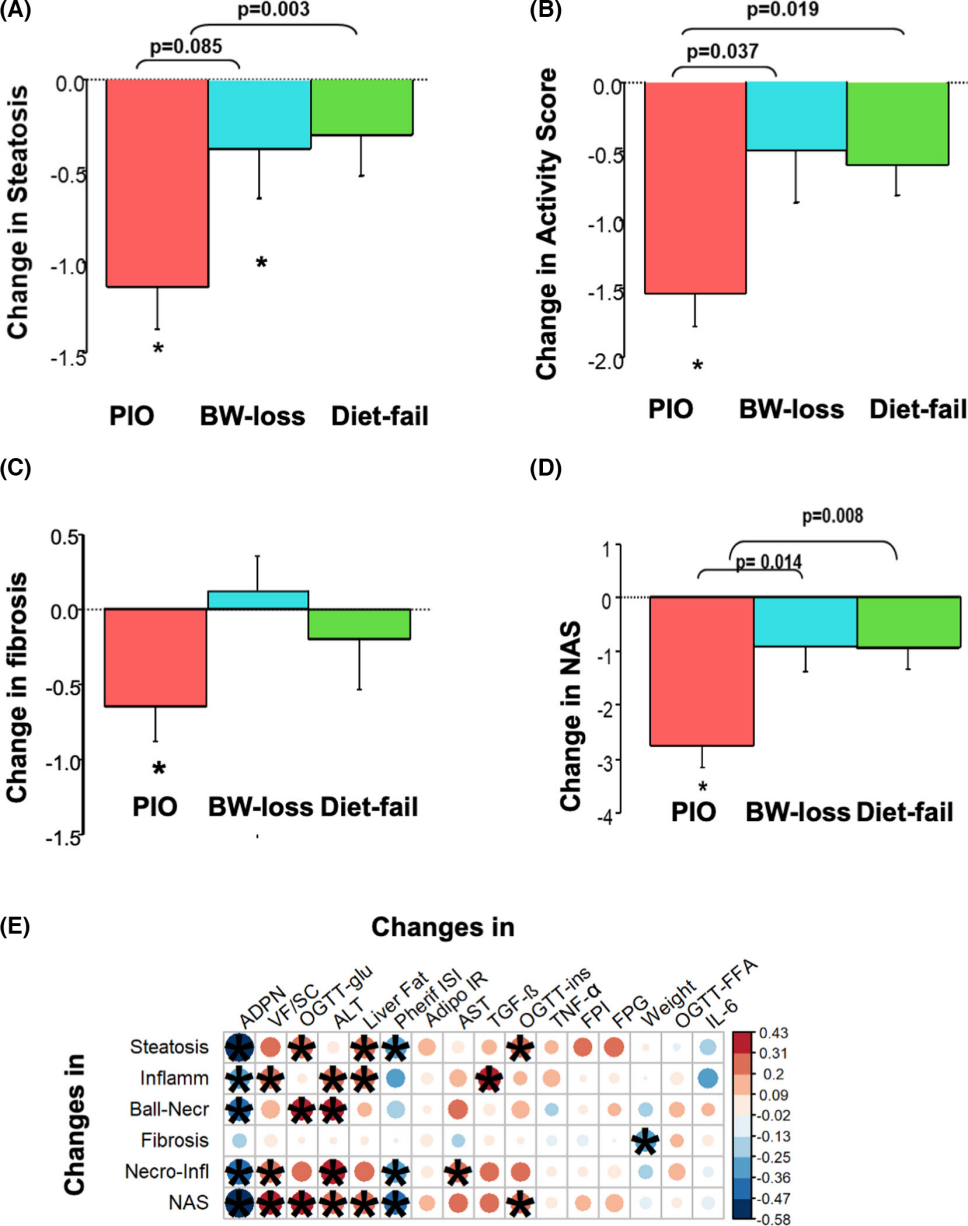
Changes (post‐pre values) in liver histological parameters in patients with NASH after pioglitazone (“PIO”; red bars), weight loss following dietary counselling (“BW‐loss”; blue bars), or dietary failure to nutritional counselling (“Diet‐fail”; green bars). Panel A: steatosis; Panel B: disease activity score (as hepatocyte ballooning and lobular inflammation); Panel C: fibrosis; and Panel D: NAS. *P < .05 vs pretreatment. Panel E: correlation matrix between changes in metabolic variables and individual liver histological parameters (steatosis, ballooning, lobular inflammation, their combined activity score or fibrosis). The size and intensity of the colour indicate the value of the Spearman correlation coefficient (according to the colour bar on the right, that is, red circles indicate a positive correlation while blue circles a negative correlation). * indicates a correlation coefficient with *P* < .05. Key variables (changes in adiponectin, VF/SC, glucose during the OGTT, ALT and liver fat by MRS; as well as weight and other metabolic parameters) have been organized from left to right based on their relative importance from the machine learning approach with PLS‐DA. Of note, PLS‐DA discriminated well the treatment effect of pioglitazone on necroinflammation, while changes in steatosis (and NAS) were less discriminatory for the effect between pioglitazone and placebo. NAS, NAFLD Activity Score; NASH, non‐alcoholic steatohepatitis; OGTT, oral glucose tolerance test; PLS‐DA, partial least square discriminant analysis; VF/SC, visceral‐to‐subcutaneous fat ratio

We found that the variables that better discriminate (by machine learning with PLS‐DA) pioglitazone compared to placebo treatment were the increase in plasma ADPN concentration and the VF/SC fat ratio (an index of abdominal fat distribution), the mean glucose concentrations during an OGTT, plasma aminotransferases (ALT) and liver fat content (41% of variability explained, VIP > 1) (Figure 3B and Figure S2A).

Although with lower accuracy, PLS‐DA was also able to discriminate well the pioglitazone vs “BW‐loss” groups (Figure S1A, Table S1), with the same parameters (a decrease in plasma ADPN concentration, ALT, mean OGTT glucose concentration and the VF/SC fat ratio, Figure S2B) being the variables with the highest discriminative power but of note changes in liver fat did not predict response (VIP < 1, Figure S1B and *P* = ns, Mann‐Whitney test).

### 3.2 Effect of treatment on liver histology

Pioglitazone‐treated patients showed a significant improvement in several histological parameters compared to the placebo‐diet intervention groups. While pioglitazone reduced NAS (*P* < .007) and fibrosis (*P* < .05), the “BW‐loss” group only reduced steatosis vs baseline, and no significant change was observed in the “Diet‐fail” group (Figure 3).

To evaluate the predictive power of changes in metabolic parameters and VF/SC fat on any change/improvement in liver histology we used PLS‐DA to discriminate subjects according to their improvements in steatosis, AS and NAS. Despite the small cohort, PLS‐DA was able to discriminate subjects that decreased hepatic fat from those without improvements in steatosis (Figure S3) with a high predictive power and statistical significance (Table S3). From this analysis the variables most relevant for the classification were the increase in plasma ADPN concentration and Periph‐ISI (calculated as OGIS), the decrease in VF/SC fat ratio and ALT concentrations (Figures S3 and S4). Although with less accuracy in prediction, PLS‐DA was also able to classify subjects that reduced disease AS (Figure S3, Table S3). Among the variables with the highest discriminative power, we found the same parameters of the model about steatosis (changes in ALT and ADPN concentration, VF/SC fat ratio and Periph‐ISI), but of note, also the reduction of TGF‐β concentration (Figure S4).

Regardless of treatment, improvements in liver histology were associated with the improvements in adipose tissue function (eg ADPN) and the most relevant metabolic variables of the PLS‐DA model. Figure 3E shows key variables, such as changes in ADPN, VF/SC, glucose during the OGTT, ALT and liver fat by MRI, that were organized based on their relative importance from the machine learning approach (VIP score, Figure 2B). Reduction in steatosis and disease AS showed a strong association with increased plasma ADPN levels (*r* = −.71 and *r* = −.44, *P* < .007, respectively) and with the reduction in VF/SC fat (*r* = .41 and *r* = .37, *P* < .03 respectively). Beyond these two parameters, the degree of improvement in hepatic lobular inflammation was also related with improvement in peripheral (liver and muscle) insulin resistance (PERIPH‐ISI) and with plasma ALT (*r* = −.38, *P* = .02 and *r* = .33, *P* < .05, respectively), while amelioration of ballooning also showed a significant correlation a reduction in mean plasma glucose during the OGTT (*r* = .37, *P* = .02), associations also proved by multivariable analysis (ie adjusted for key variables).

### 3.3 **Effect of treatment on adipose tissue function and distribution and** IR

**FIGURE 4:**
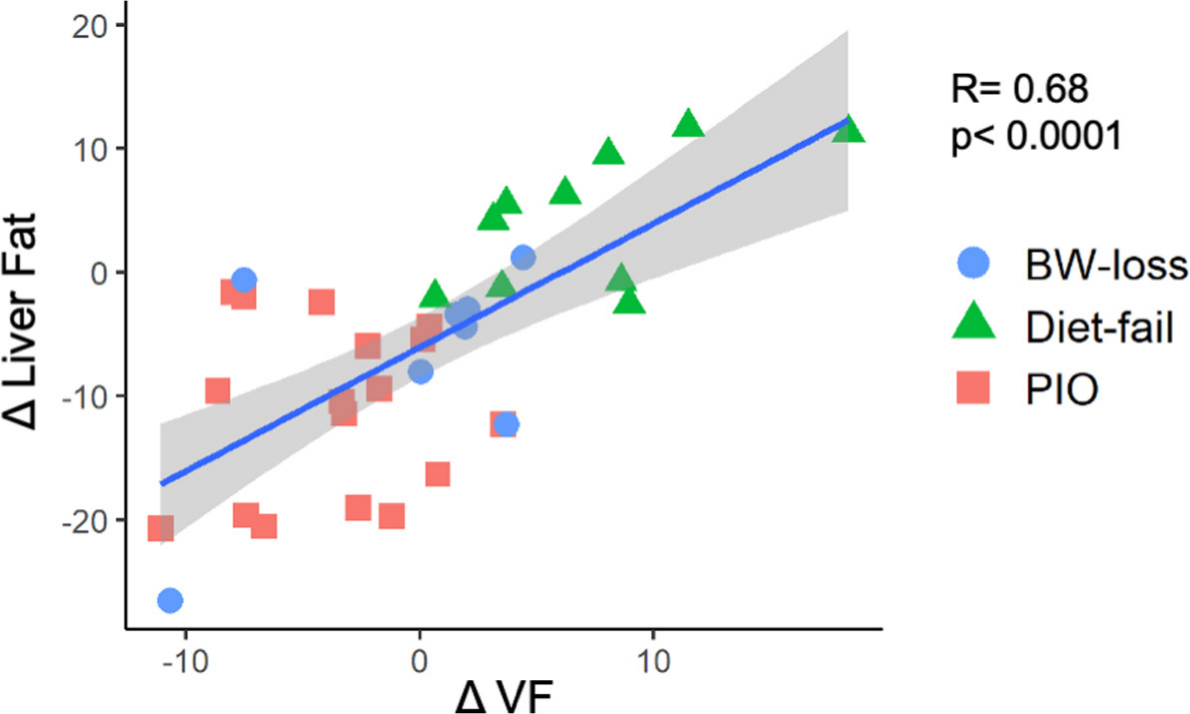
Univariate regression analysis in the entire cohort of NASH patients showed strong association between changes in liver fat with respect to VF. Patients treated with pioglitazone (“PIO” red squares) were those with the strongest decrease in both VF and hepatic fat. In placebo group those that did not lose weight (“Diet‐fail”; green triangles) and those that lost weight (“BW‐loss”; blue circles) showed the same association between changes in VF and liver fat. NASH, non‐alcoholic steatohepatitis; VF, visceral fat

We found that not only VF and liver fat were highly correlated before treatment (*r* = .45, *P* = .009) but also changes in liver fat were strongly correlated with changes in VF (*r* = .68, *P* < .0001) in all patients regardless of treatment (Figure 4). However, patients treated with pioglitazone (“PIO” red squares) were those with the strongest decrease in both hepatic and VF while among patients in the placebo groups, those that did not lose weight (“Diet‐fail”; green triangles) increased both VF and hepatic fat, and among those that lost weight (“BW‐loss”; blue circles) only those that decreased VF had a reduction in hepatic fat.

**FIGURE 5:**
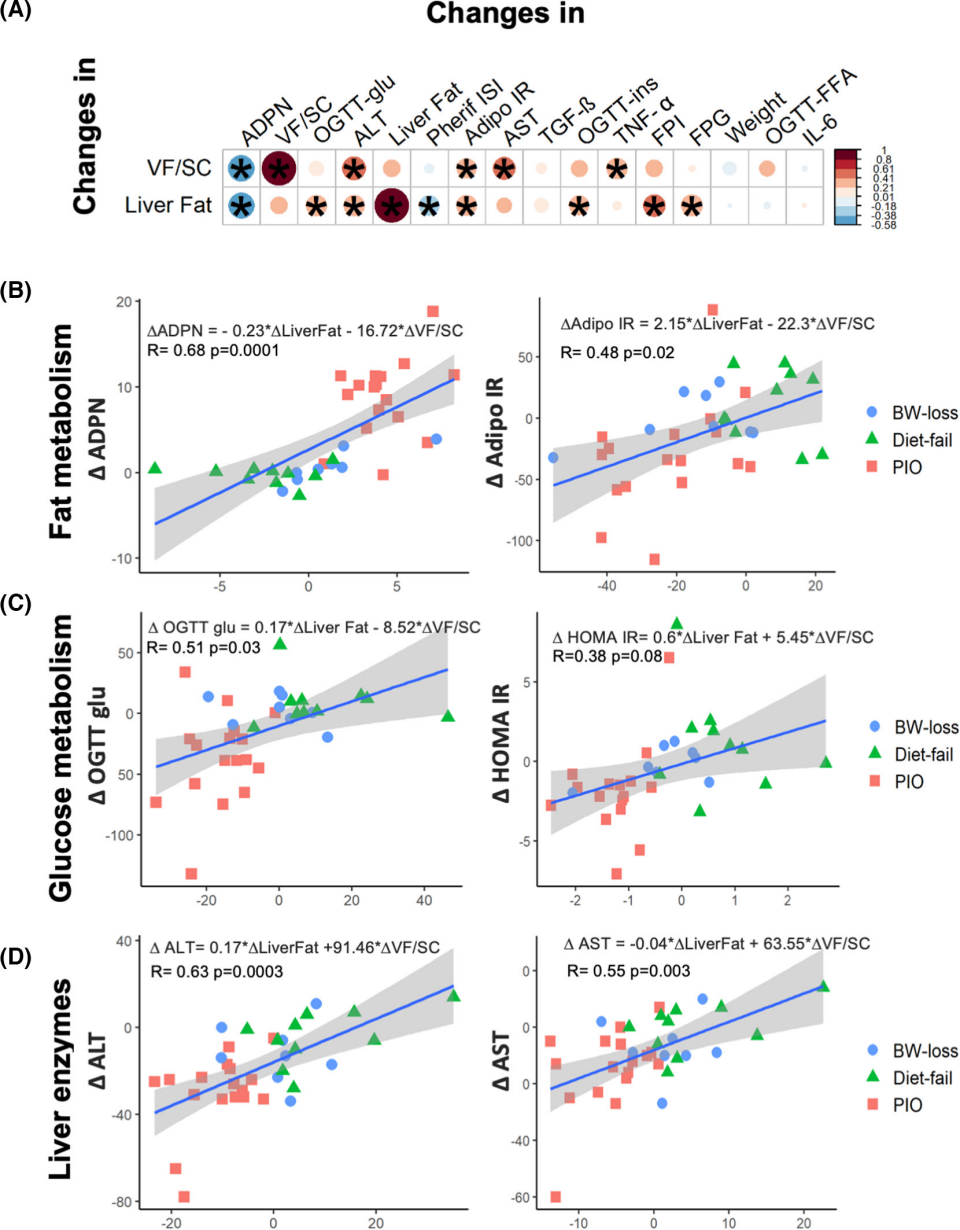
Panel A: Correlation matrix showing the univariate correlation between changes (post‐ pre) in intrahepatic TG (liver fat) and VF/SC ratio and changes in metabolic parameters in the entire cohort. The size and intensity of the colour indicate the value of the Spearman correlation coefficient (according to the colour bar on the right, that is, red circles indicate a positive correlation while blue circles a negative correlation). *P < .05. Key variables (changes in adiponectin, VF/SC fat, glucose during the OGTT, ALT and liver fat by MRS; as well as weight and other metabolic parameters) have been organized from left to right based on their relative importance from the machine learning approach with PLS‐DA showed in Figure 2. Multiple regression analysis for changes in plasma adiponectin and adipose tissue insulin resistance (Adipo‐IR) (Panel B); HOMA and mean glucose excursions during an OGTT (Panel C); plasma ALT and plasma AST (Panel D), with respect to change in liver fat and VF/SC fat ratio, in patients with NASH that lost weight following dietary counselling (“BW‐loss”; blue circles), fail the diet (“Diet‐fail”; green triangles) or were treated with pioglitazone (“PIO” red squares). OGTT, oral glucose tolerance test; PLS‐DA, partial least square discriminant analysis; VF/SC, visceral‐to‐subcutaneous fat ratio

We then explored if changes in liver fat or VF/SC were related to each other and to changes in adipose tissue function and tissue insulin sensitivity. Figure 5A shows the univariate correlation matrix between liver, and VF/SC fat ratio with a broad spectrum of metabolic variables, ordered in accordance with their relative importance from PLS‐DA model (Figure 2). Of note decrease in liver fat was associated with the decrease in VF/SC fat, adipo‐IR and the increase in ADPN and peripheral IR. No significant association was found between the changes in liver fat and changes in total SC fat or changes in BW or changes in TGF‐β and TNF‐α (Figure 5A).

Changes in both VF/SC fat ratio and liver fat were correlated with the increase in ADPN (*r* = −.70 and *r* = −.63, *P* < .0001, respectively), the decrease in Adipo‐IR (*r* = .44 and *r* = .48, *P* < .008, respectively), HOMA‐IR and mean glucose during OGTT and the decrease in liver enzymes levels and the decrease in TNF‐α was associated to the decrease in VF/SC fat and VF (*r* = .38, *P* = .009, and *r* = .32, *P* = .02, respectively), while the decrease in TGF‐β was associated to the increase in SC fat (*r* = −.35, *P* = .02, Figure 5A).

The univariate associations of the improvements in lipid and glucose metabolism and the reduction in liver enzymes with both hepatic and VF/SC fat ratio were also confirmed by multivariable linear regression models (Figure 5B‐D), regardless of treatment.

Modest weight loss in the BW‐loss group (~3%‐4%) was insufficient to either decrease fasting FFA, improve adipose tissue insulin resistance (Adipo‐IR) or increase plasma ADPN concentration (Table 1). Similar results were observed in the Diet‐Fail group. In contrast, patients treated with pioglitazone showed a significant improvement in both parameters of adipose tissue function (Table 1). ADPN concentrations increased only with pioglitazone but not after weight loss (Table 1).

Regardless of treatment, we found that an increase in plasma ADPN concentration was associated to the decrease in Adipo‐IR (*r* = −.70, *P* = .0002) as well as increase in Hep‐ISI (*r* = .53, *P* = .003). Changes in TNF‐α were negatively associated with ADPN (*r* = −.35, *P* = .02).

The Hep‐ISI was similar in all groups at baseline (Table 1). In the “BW‐loss” group Hepatic‐IS increased but it did not reach significance, while in the pioglitazone group we observed a significant increase (*P* < .02 vs baseline and Diet‐fail). Consistent with the above results, Periph‐ISI was unchanged by diet only, but improved significantly in the pioglitazone group (Table 1). The improvement in Hep‐ISI was correlated with the decrease in VF (*r* = −.42, *P* = .01) and liver fat (*r* = −.37, *P* < .03), but not with changes in SC fat (*r* = .07, *P* = ns).

## 4 DISCUSSION

The mechanisms responsible for the decrease in liver fat and overall improvement in steatohepatitis are unclear. NAFLD is often associated with obesity and IR.^15^, ^43^ However, it is not the amount of BW and overall fat, but the severity of adipose tissue dysfunction/IR,^9^, ^17^ and its distribution,^2^, ^4^, ^44^ that appear to be more important determinants of the alterations of glucose and lipid metabolism and responsible of end‐organ lipotoxicity. VF is more metabolically harmful than SC fat^2^, ^3^, ^4^, ^15^, ^43^, ^44^ and quantification of VF is important not only because it can promote NAFLD, but also because VF may play a role in the progression to NASH and cirrhosis,^7^ and is directly implicated in the risk and progression of several gastrointestinal cancers.^45^ Prior studies in patients treated with PPAR‐γ agonists have shown that, despite an increase in SC fat and BW, their metabolic status was improved by treatment and more in the treatment group achieved resolution of NASH.^20^, ^22^, ^23^, ^30^, ^31^ By using a machine learning approach based on PLS‐DA analysis, we investigated if the improvement in adipose tissue function (eg by increase in ADPN and changes in inflammatory markers), the changes in body fat distribution and the reduction in VF, by either weight loss or a PPAR‐γ agonist (pioglitazone), could explain the improvement in liver histology in NASH.

A key finding is that VF and liver fat were highly correlated at baseline, in agreement with previous studies,^9^, ^10^, ^11^ and that a PPAR‐γ agonist can decrease VF, liver fat and steatohepatitis disease activity despite a general increase in total body fat. In contrast, in the BW‐loss group only liver fat decreased, but not necroinflammation, and its failure was associated with no change in VF while in the Diet‐fail group a slightly BW increase was related with a disproportionate expansion of VF.

In our cohort of subjects with prediabetes or diabetes the decrease in liver fat, either by pioglitazone or placebo with weight loss (Figure 1D), was positively correlated with the decrease in VF independent of a small, but significant, increase in BW and total body fat in the pioglitazone group (Figure 1A). This is in agreement with the "portal hypothesis" and enhanced portal fatty acids delivery to the liver.^46^ Our data confirmed that it is not total fat but rather changing the biology of fat (ie improving adipose tissue IR) and its location (Figure 1) that is important. Thus, weight gain by pioglitazone, limited largely to gain in SC fat but with a reduction in VF, lacks the negative impact on hepatic fat and necroinflammation observed from weight gain under conditions of chronic overnutrition.

Another important result of this study is regarding the changes in liver histology observed after placebo plus dietary intervention vs pioglitazone. In the BW‐loss group, steatosis on imaging was significantly improved (Figure 1D), but less than in the pioglitazone treated group (Figure 1A), while the disease activity and NAS were changed only in the pioglitazone group (Figure 3B,D respectively). On the other hand, the dietary counselling even when successful for weight‐loss, was modest in this group and not sufficient to improve necroinflammation or fibrosis as much as pioglitazone (Figure 3B,C, respectively), in agreement with previous studies.^31^, ^39^ This is not surprising since many factors, beside weight loss, contribute to the improvement of liver fibrosis, as also shown by the recent 72‐weeks trial with semaglutide that did not improve fibrosis despite a 13% weight loss.^47^

The decrease in VF/SC distribution and the increase in ADPN play an important role in mediating the histological effect of pioglitazone and were strongly associated with the decrease in AS and NAS (Figure 3E). VF is a major source of pro‐inflammatory cytokines like IL‐6^6^, ^46^ and ADPN concentrations are usually decreased in subjects with high VF and in relation to reduced insulin sensitivity.^6^ Pioglitazone is associated both with fat re‐distribution from visceral to SC tissue and with a reduction in pro‐inflammatory (eg IL‐6) and an increase in anti‐inflammatory (eg ADPN) adipokines.

It has been suggested that hepatic steatosis in NAFLD is the result of the increased lipolysis (mainly from SC adipose tissue) and delivery of FFAs to the liver.^5^, ^15^, ^43^ One of the mechanisms for the improvement in liver fat and liver histology after PPAR‐γ agonists is the reduction of IR in particular in the adipose tissue.^25^, ^26^ Treatment with PPAR‐y agonists improves both peripheral^13^, ^24^ and adipose tissue^25^, ^26^, ^27^ IR. Circulating FFA and Adipo‐IR were decreased only in the pioglitazone group (Table 1), in agreement with previous studies that showed decreased peripheral lipolysis and improvement in the antilipolytic effect of insulin after pioglitazone treatment.^25^, ^27^ Although the contribution of VF lipolysis to systemic FFA is probably minimal^46^ the great majority of FFA secreted by VF are released into the portal vein and thus taken up by the liver on their first pass. Both the decrease in VF and liver fat correlated with the improvement in Hep‐ISI (Figure 5A), in agreement with data previously published showing that increased VF is associated with increased gluconeogenesis and IR^1^, ^9^, ^12^ and that after treatment with PPAR‐γ agonists the decrease in gluconeogenesis was proportional to the decrease in VF content despite the increase in BW.^28^, ^29^ In this study, we also evaluated the association between changes in both liver fat and VF and changes in hepatic and peripheral insulin sensitivity, in the whole cohort and separately in the pioglitazone and placebo groups. In the whole cohort we found that the improvement in Hep‐ISI was proportional only to the reduction in both hepatic and VF (Figure 5A), but not changes in SC or total fat. Peripheral insulin sensitivity during the OGTT (OGIS) was unchanged by diet and only improved significantly in the pioglitazone treated group (Table 1).

Adiponectin is one of the most important adipose tissue released anti‐inflammatory adipokine that can signal in the liver. ADPN affects hepatic glucose and lipid metabolism, since it stimulates fatty acid oxidation and inhibit hepatic fatty acid synthesis via activation of AMP‐activated protein kinase. Patients with NAFLD usually display low plasma ADPN concentrations compared to matched‐patients without NAFLD or with isolated steatosis.^22^, ^48^, ^49^, ^50^ PPAR‐γ agonists improve Adipo‐IR by reducing lipolysis and increasing ADPN^25^, ^26^, ^27^, ^28^ and it has been suggested that ADPN may play an important role in mediating the beneficial effects of pioglitazone.^22^, ^28^, ^50^ In this study, ADPN was increased by 2‐ to 3‐fold above baseline values, but only after pioglitazone and not after weight loss (Table 1). Weight loss by lifestyle intervention has a modest effect on ADPN in patients with T2D as illustrated in the Look AHEAD study, where a ~10% weight loss increased plasma ADPN by only 12%.^51^ A significant (≥1.5‐fold) increase in plasma ADPN requires a much larger weight loss, as observed after bariatric surgery,^52^ but still the change is less than that observed with PPAR‐γ agonists in patients with NAFLD.^22^, ^31^, ^49^, ^50^ A significant correlation was observed between the increase in plasma ADPN levels and the reduction of both liver and VF content (Figure 5B), independently of type of treatment. The changes in plasma ADPN levels observed in this study were associated with improvement in both hepatic and adipose tissue IR, in agreement with previous results also from our group that showed that changes in ADPN after pioglitazone were followed by a reduction in both hepatic IR and gluconeogenesis.^13^, ^28^ More recently, we have reported that an increase in plasma ADPN concentration is likely to play an important role for the long‐term histological benefit of pioglitazone.^31^

In summary, these data show that VF accumulation is strongly associated to liver fat, ballooning and lobular inflammation and that most of the improvements in metabolic/histological parameters observed after pioglitazone are strongly associated with changes in adipose tissue distribution and biology (ie ADPN, lipolysis and IR). In conclusion, changes in both VF and plasma ADPN release play an important role in mediating the beneficial effects of PPAR‐γ agonist treatment in patients with NASH.

## DISCLOSURES

S.S, CF, GM, FB, RA, PV, DB, SK have nothing to disclose.

AG has received honorarium from Novo Nordisk and is consultant for Boehringer Ingelheim, Eli Lilly, Gilead, Inventiva, and Pfizer.

KC has received research support as principal investigator for the University of Florida from the National Institute of Health (NIH), Cirius, Echosens, Inventiva, Novartis, Novo Nordisk, Poxel and Zydus. KC is also a consultant for Allergan, Altimmune, Arrowhead, AstraZeneca, BMS, Boehringer Ingelheim, Coherus, Eli Lilly, Fractyl, Hanmi, Genentech, Gilead, Intercept, Janssen, Pfizer, Prosciento, Madrigal and Novo Nordisk.

SAH: Scientific advisor or consultant for Akero, Alentis, Altimmune, Arrowhead, Axcella, Canfite, Cirius, CiVi Biopharma, Cymabay, Echosens, Fibronostics, Forest Labs, Galectin, Genfit, Gilead, Hepion, HistoIndex, Intercept, Madrigal, Medpace, Metacrine, NGM Bio, Northsea, Novartis, Novo Nordisk, PathAI, Poxel, Liminal, Ridgeline, Sagiment, Terns, Viking, 89 Bio. Stock options: Akero, Cirius, Galectin, Genfit, Hepion, HistoIndex, PathAI, Metacrine, NGM Bio, Northsea. Grant/Research support: Akero, Axcella, BMS, Cirius, CiVi Biopharma, Conatus, Cymabay, Enyo, Galectin, Genentech, Genfit, Gilead, Hepion, Hightide, Intercept, Madrigal, Metacrine, NGM Bio, Novartis, Novo Nordisk, Northsea, Pfizer, Sagimet, Viking.

## Supporting information

Supplementary Material
