# Supplementary material for: PPAR‐γ‐induced changes in visceral fat and adiponectin levels are associated with improvement of steatohepatitis in patients with NASH
Source: Liver Int. 2021 Jul 21;41(11):2659–70. doi: 10.1111/liv.15005 (PMC9290929; doi:10.1111/liv.15005)
Supplement: Supplementary file 1 — Supplementary Material [file LIV-41-2659-s001.docx]

# Supplementary Materials

**Study design.** A total of fifty-five participants were enrolled and seven withdrew during the study (one in the run-in; six within 9 weeks of treatment) ([22](#_ENREF_22)). Some patients were unable to complete both the pre- and post-liver fat and visceral fat MRI for this analysis due to factors claustrophobia, inability to fit in the MRI scanner due to severe central obesity or scheduling issues (n=12). After baseline metabolic measurements, patients were randomized to either oral placebo or pioglitazone (ACTOS^®^, Takeda Pharmaceuticals) 30 mg/day, titrated after 2 months to 45 mg/day until the end of the 6-month study (see Consort flow diagram in supplementary material). The cohort included both patients with type 2 diabetes or glucose intolerance diagnosed by an oral glucose tolerance test (OGTT) performed at baseline and end of study (see below). All patients were educated at the beginning of the study by the research dietician to reduce their intake by -500 kcal per day and continued during follow-up visits ([22](#_ENREF_22)). Liver biopsies were scored according to Kleiner et al ([38](#_ENREF_38)).


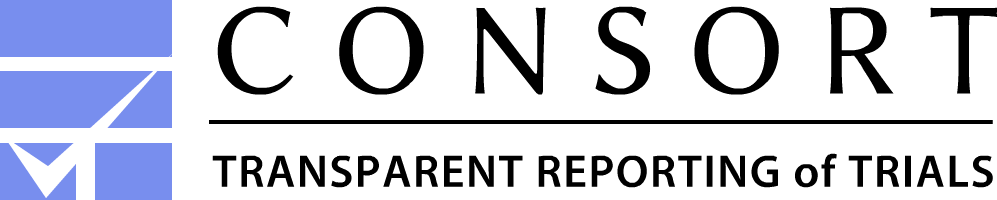


**CONSORT 2010 Flow Diagram**

Analysed (n= 17 )
♦ Excluded from analysis (give reasons) (n=8)

- Missing MRI data for abdominal fat (n=8)

Lost to follow-up (give reasons) (n= 4)

- Compliance with study medication (n=1)
- Discontinued intervention (give reasons) (n= 3)
- Personal reason (n=1)
- Suspected CAD (n=1)
- Fatigue plus edema (n=1)

Lost to follow-up (give reasons) (n= 4)

- Discontinued intervention (give reasons) (n= 4)
- Altered liver aminotransferase levels (n=1)
- Personal reason (n=1)
- Suspected CAD (n=1)
- Fatigue (n=1)

Analysed (n= 18)
♦ Excluded from analysis (give reasons) (n=3)

- Missing MRI data for abdominal fat (n=3)

## Analysis

Allocated to Pioglitazone (n= 30 )

♦ Received allocated intervention (n= 30 )

♦ Did not receive allocated intervention (give reasons) (n= 0)

## Follow-Up

## Enrollment

## Allocation

Allocated to placebo (n= 25)

♦ Received allocated intervention (n= 25)

♦ Did not receive allocated intervention (give reasons) (n= 0)

Randomized (n= 55)

Excluded (n= 15 )

♦  Not meeting inclusion criteria (n= 15 )

♦  Declined to participate (n= 0 )

♦  Other reasons (n= 0 )

Assessed for eligibility (n= 70)

Partial Least Square Discriminant Analysis (PLS-DA) was used to discriminate the effect of PIO vs body weight loss (BW-loss), using all metabolic (log2 post/pre) and histological (post-pre) variables (**Figure S1**, **S2** and **Table S1 and S2**). We have evaluated PIO vs Placebo, but also PIO vs BW-loss, PIO vs Diet fail, or PIO vs BW-loss and Diet fail. In all these classifications, PLS-DA achieved high accuracies.

**Figure S1.** Panel A: Scores plot of PLS-DA in PIO (red points) vs BW-loss (blue points) subjects’ classification. Panel B: Variables contribution to the PLS-DA model, measured through VIP index. Features with VIP>1 are considered relevant in the discrimination.

**Table S1** shows the PLS-DA models' hyperparameters (number of components), performance in prediction (accuracy) and statistical significance (p-value) based on treatment and outcome (PIO vs Placebo, PIO vs Placebo with BW loss, PIO vs Placebo with Diet fail, PIO vs BW-loss and vs Diet-fail). **Table S2** lists the features used in PLS-DA models in the discriminations PIO vs Placebo (**Figure 2**) and PIO vs BW-loss(**Figure S1**) and relative Mann-Whitney test’s p-values.

**Table S1.** Performance of PLS-DA models for PIO vs Placebo or placebo subgroups

**Table S3.** P values for variables used in PLS-DA models (PIO vs placebo or vs BW-loss)

**Figure S2** shows the variables (expressed as log2 of fold changes) that mostly contributed to the PLS-DA model of PIO vs placebo (Panel A) or of PIO vs BW-loss subgroup (Panel B)


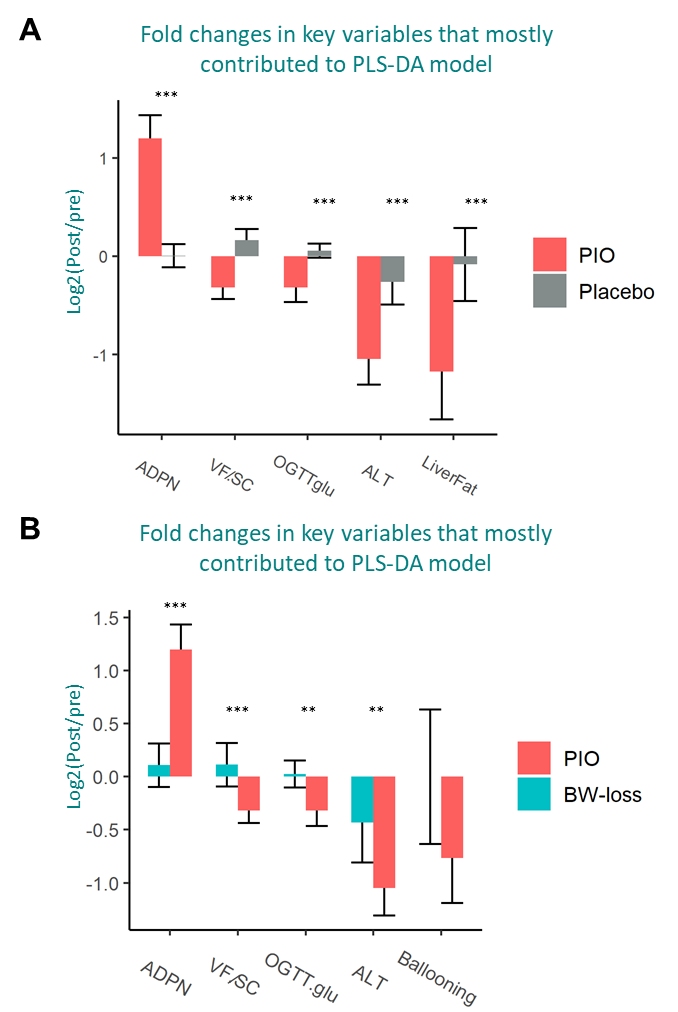


**Figure S2.** Panel A: barplot of variables that are relevant in the discrimination PIO (red bars) vs PLACEBO (grey bars) in the PLS-DA model (VIP>1). Panel B: barplot of variables that are relevant in the discrimination PIO (red bars) vs BW-loss (blue bars) in the PLS-DA model (VIP>1). ***: p-value< 0.001, **: p-value< 0.01, *: p-value < 0.05.

Partial Least Square Discriminant Analysis (PLS-DA) was also used to discriminate subjects according to their improvements in histology, using all metabolic (log2 post/pre) variables. We have evaluated steatosis (**Figure S3**), activity score (AS) (**Figure S4**) and NAS score (**Table S3**) on the entire cohort. PLS-DA achieved high accuracies and statistical significances in the classification models.

**
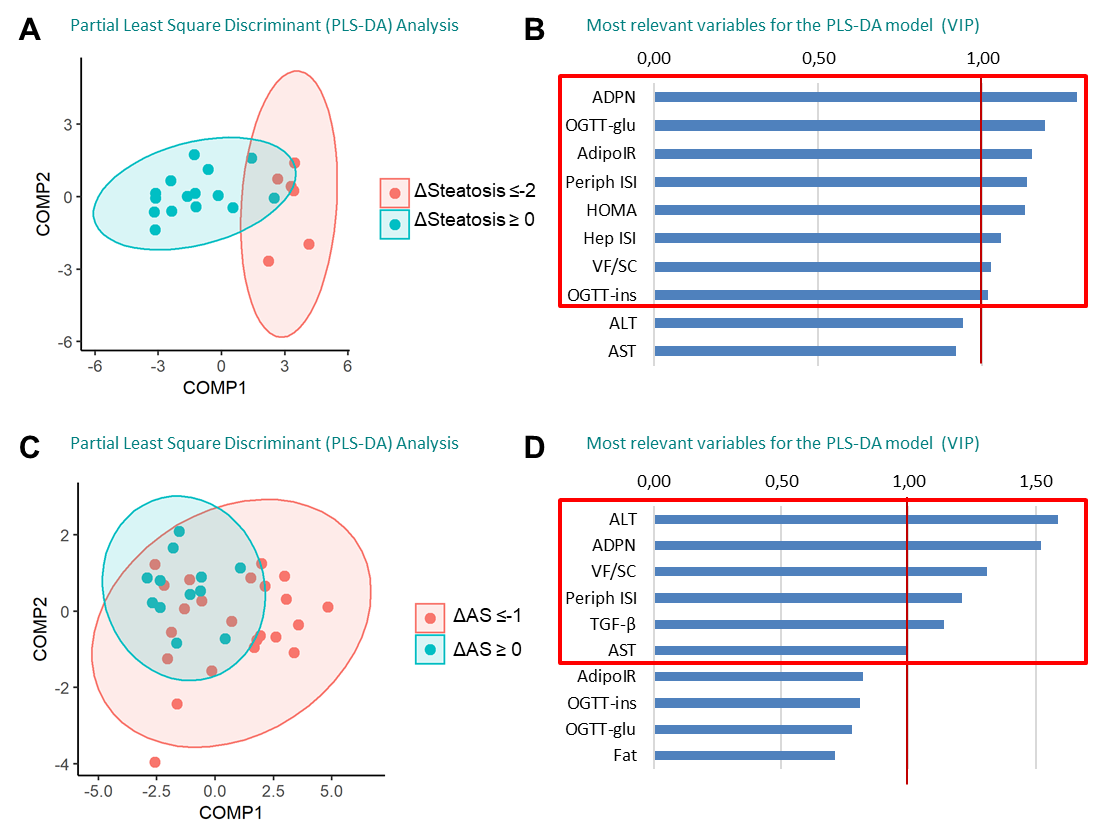
**

**Figure S3. Panel A:** Subjects were grouped according to changes in steatosis score (decrease of at least 2 points, red dots, vs an increase, blue dots). **Panel B:** Variables contribution to the PLS-DA model on steatosis, measured through VIP index. **Panel C:** Subjects were then grouped according to changes in activity score (decrease of at least 1 point, red dots, vs an increase, blue dots). **Panel D:** Variables contribution to the PLS-DA model on activity score, measured through VIP index.

**Table S3** shows PLS-DA models' hyperparameters (number of components), performance in prediction (accuracy) and statistical significance (p-value) based on histological classifications (steatosis, activity score, AS, and NAS score). **Table S4** shows the list of features used in PLS-DA models on changes in steatosis and activity score (**Figure S3**) and relative Mann-Whitney test’s p-values.

**Table S3.** Performance of PLS-DA models for changes in steatosis, activity score and NAS score


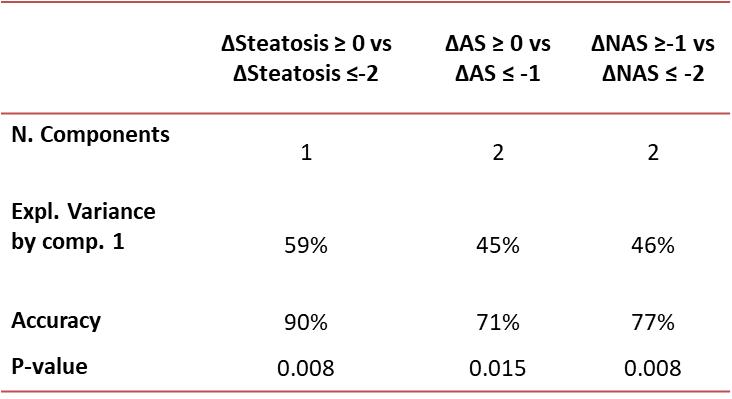


**Table S4.** P values for variables used in PLS-DA models (changes in steatosis or changes in activity score)

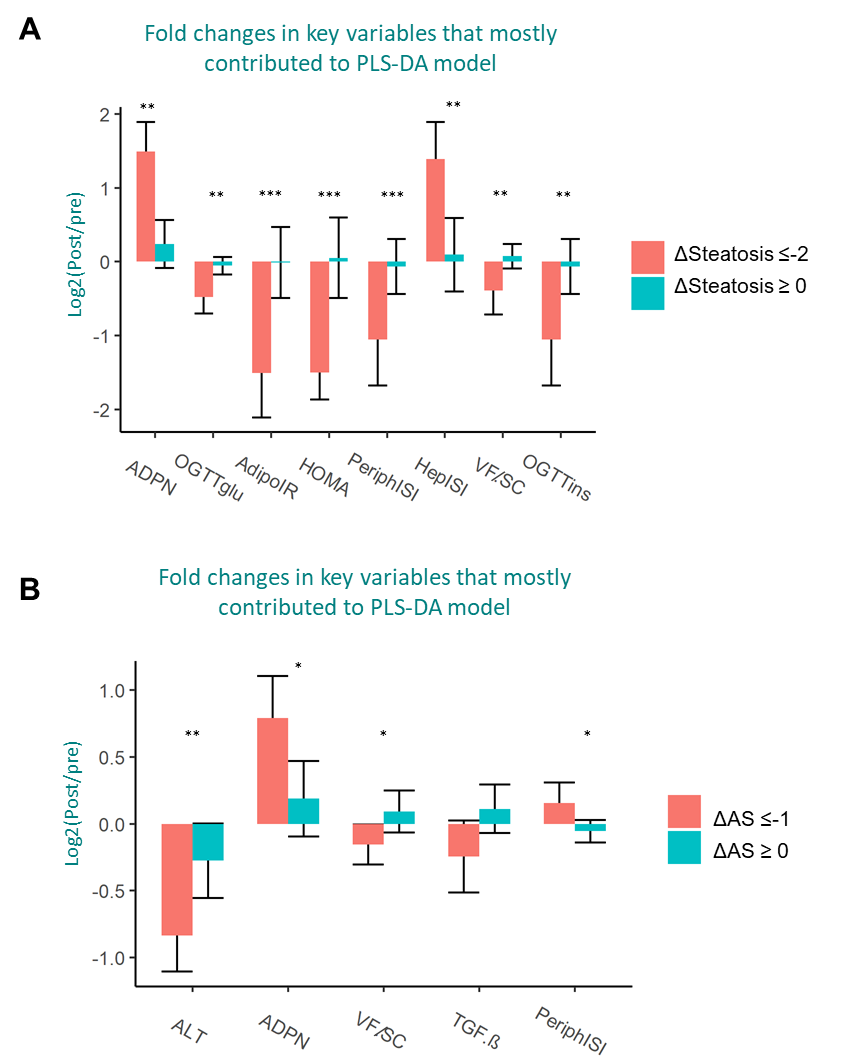


**Figure S4.** **Panel A**: barplot of variables that are relevant in the discrimination for the PLS-DA model (VIP>1) on changes in steatosis score (decrease of at least 2 points, red bars, vs an increase, blue bars). **Panel B**: barplot of variables that are relevant in the discrimination for the PLS-DA model (VIP>1) on changes in activity score (decrease of at least 1 point, red bars, vs an increase, blue bars). ***: p-value< 0.001, **: p-value< 0.01, *: p-value < 0.05.

**List of abbreviations:**

Adipo IR: adipose tissue IR index

ADPN: adiponectin

ALT: alanine transaminase

AST: aspartate transaminase

AS: activity score

NAS: NAFLD activity score

Ballooning: Ballooning score in liver biopsy

Fat

Fibrosis

Hep ISI: hepatic insulin sensitivity index

HOMA: homa IR index

IL-6: interleukin 6 concentration

Inflammation

Liver Fat

OGTT-glu: mean glucose concentration during OGTT

OGTT-ins: mean insulin concentration during OGTT

Periph ISI: peripheral insulin sensitivity index calculated as OGIS

Steatosis:

TGF-β: transforming growth factor-β concentration

TNF-α: tumor necrosis factor-α concentration

VF/SC: VF-to-subcutaneous fat ratio
